# The Trapping Mechanism at the AlGaN/GaN Interface and the Turn-On Characteristics of the p-GaN Direct-Coupled FET Logic Inverters

**DOI:** 10.3390/nano14241984

**Published:** 2024-12-11

**Authors:** Junfeng Yu, Jihong Ding, Tao Wang, Yukai Huang, Wenzhang Du, Jiao Liang, Hongping Ma, Qingchun Zhang, Liang Li, Wei Huang, Wei Zhang

**Affiliations:** 1 State Key Laboratory of ASIC and System, Shanghai Institute of Intelligent Electronics & Systems, School of Microelectronics, Fudan University, Shanghai 200433, China; 2 East China Institute of Photo-Electron IC, Bengbu 233000, China; dingjihong2005@163.com; 3 National Key Laboratory of Integrated Circuits and Microsystems, Wuxi 214035, China; wanty678@126.com; 4 Institute of Wide Bandgap Semiconductors and Future Lighting, Academy for Engineering & Technology, Fudan University, Shanghai 200433, China; 5 School of Electronic Information Engineering, Suzhou Vocational University, Suzhou 215104, China

**Keywords:** DCFL inverters, trapping mechanism, p-GaN HEMTs

## Abstract

The trapping mechanism at the AlGaN/GaN interface in the p-GaN high electron mobility transistors (HEMTs) and its impact on the turn-on characteristics of direct-coupled FET logic (DCFL) inverters were investigated across various supply voltages (*V*_DD_) and test frequencies (*f*_m_). The frequency-conductance method identified two trap states at the AlGaN/GaN interface (trap activation energy *E*_c_-*E*_T_ ranges from 0.345 eV to 0.363 eV and 0.438 eV to 0.47 eV). As *V*_DD_ increased from 1.5 V to 5 V, the interface traps captured more electrons, increasing the channel resistance (*R*_channel_) and drift-region resistance (*R*_drift_) of the p-GaN HEMTs and raising the low-level voltage (*V*_OL_) from 0.56 V to 1.01 V. At *f*_m_ = 1 kHz, sufficient trapping and de-trapping led to a delay of 220 µs and a *V*_OL_ instability of 320 mV. Additionally, as *f*_m_ increased from 1 kHz to 200 kHz, a positive shift in the threshold voltage of p-GaN HEMTs occurred due to the dominance of trapping. This shift caused *V*_OL_ to rise from 1.02 V to 1.40 V and extended the fall time (*t*_fall_) from 153 ns to 1 µs. This investigation enhances the understanding of DCFL GaN inverters’ behaviors from the perspective of device physics on power switching applications.

## 1. Introduction

Wide bandgap semiconductors (WBGSs) have attracted significant attention due to their exceptional electrical, thermal, and mechanical properties, showing considerable promise in various industries and scientific domains [1]. SnO_2_ has been found to be an excellent transparent thermoelectric material [2,3]. Additionally, AlN photovoltaics have been reported to achieve high power conversion efficiency [4,5]. Compared to these ultra-wide bandgap semiconductors, Gallium Nitride (GaN) is more mature and promising in academic research and industrial development.

GaN excels in high-power, high-frequency, and high-temperature applications, owing to wide bandgap and high critical breakdown voltage [6]. GaN-based devices, especially High Electron Mobility Transistors (HEMTs), operate at higher voltages and frequencies than traditional silicon-based devices, making them ideal for power electronics, RF amplification, and optoelectronics. GaN’s high electron mobility and saturation velocity boost its performance in RF technologies. It is also promising for electric vehicle power trains, wireless charging, and energy-efficient power converters due to its high power density and compact size [7,8,9]. In recent years, investigations have been conducted on novel GaN-based devices and high-switching-speed monolithic integrated circuits. Due to the lack of complementary devices as seen in silicon CMOS technology, GaN-based monolithic integrated circuits primarily employ direct-coupled field-effect transistor logic (DCFL) units [10,11], prompting extensive research on the electrical performances of GaN DCFL devices. For example, GaN circuits based on enhancement/depletion-mode (E/D-mode) integration exhibit superior voltage swing and noise tolerance compared to those based on enhancement/enhancement-mode (E/E-mode) at higher supply voltages [12]. This capability renders E/D-mode circuits more suitable for applications requiring high speed and drive current. Furthermore, several studies have examined their high-temperature performances, demonstrating that despite reduced electron mobility at increased temperatures, GaN DCFL devices can remain operational up to 250–500 °C [13,14,15,16].

Although GaN devices have advantages in power switching, using them in DCFL units still brings challenges. One of the biggest problems with GaN DCFL devices is the high static power consumption [17], due to the always-conducting D-HEMTs with gate and source terminals being shorted, which is limited to driver-stage applications. And the dynamic degradation induced by traps is also a concern [6]. In practice, defect behaviors can vary with changes in external bias. For instance, higher supply voltages (*V*_DD_) can intensify electron trapping, leading to current collapse [18,19]. In particular, GaN-based transistors are highly sensitive to hard switching operation, which induces dynamic threshold voltage shifts and on-resistance degradation [20]. Similarly, as the operating frequency (*f*_m_) increases, devices drain the current and the switching speed declines due to the dominance of trapping, resulting in degrading performances [21,22]. In addition, the high dependence of device performance on process consistency also increases the difficulty of achieving large-scale integration [23]. However, these studies either focus on the defect mechanism of a discrete device or observe the transformation of DCFL device performance from a more macroscopic perspective, and, rarely, the defect mechanism is closely connected with the degradation of the unit circuit. Accordingly, an in-depth study of trap mechanisms at the circuit level is crucial for optimizing GaN device performance.

The impact of AlGaN/GaN interface traps in p-GaN high-electron-mobility transistors (HEMTs) on DCFL inverters’ performances is investigated firstly in this paper. Through interface trap characterization and two-dimensional numerical simulations, the trapping mechanism under varying *V*_DD_ and *f*_m_ is discussed, with corresponding analyses of their influence on DCFL inverters’ turn-on characteristics, in order to explore the correlation between the trapping mechanism and the degradation of GaN unit circuits.

## 2. Device Fabrication and Characterization

**Figure 1:**
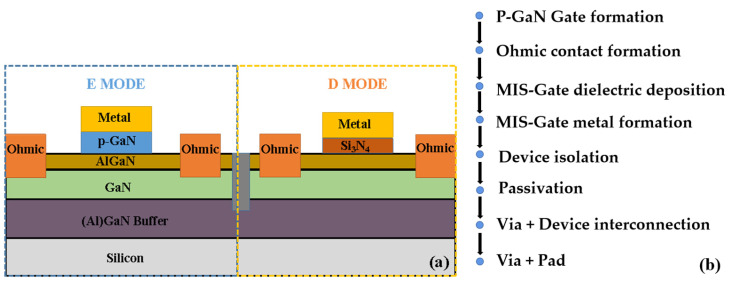
(**a**) Cross-sectional schematic of the monolithic integrated E/D-mode HEMT. (**b**) The fabrication process of E/D HEMTs.

The E/D-mode HEMTs were grown on a silicon substrate, as illustrated in Figure 1a. The device structure consists of a 15 nm Al_0.15_Ga_0.85_N barrier layer, a 200 nm GaN layer, and a 4 μm buffer layer. In the gate region, the E-mode HEMTs and D-mode HEMTs incorporate a 75 nm Mg-doped p-GaN layer with a concentration of 2 × 10^19^ cm^−3^ and a 15 nm Si_3_N_4_ insulating layer, respectively. In the inverter of this study, the E-mode HEMTs, serving as drivers, and D-mode MIS-HEMTs as active loads, are configured with *L*_GD,E_ = *L*_GS,E_ = 1.5 μm, *W*_G,E_/*L*_G,E_ = 50/1 µm/µm, and *L*_GD,D_ = *L*_GS,D_ = 2 μm, *W*_G,D_/*L*_G,D_ = 7.5/1.5 µm/µm, respectively. To effectively extract interface trap information, p-GaN stack capacitors were fabricated with the source and drain shorted, featuring a round gate area with a diameter of 200 μm.

The fabrication process of E/D HEMTs is shown in Figure 1b. Firstly, the TiN gate metal is deposited on the p-GaN layer by electron beam evaporation. Then, the p-GaN gate is formed by selective inductively coupled plasma (ICP) dry etching [24]. The process is implemented in two stages. (Stage 1: The flow rates of Cl_2_/BCl_3_ is 4/10 sccm, the chamber pressure is 1.35 mtorr, the power is 350 W, and the radio frequency (RF) power is 50 W. Stage 2: The flow rate of Cl_2_/BCl_3_ is 4/10 sccm, the chamber pressure is 1.35 mtorr, the power is 100 W, and the RF power is 10 W.) Next, a metal stack of a Ti/Al/Ni/Au layer is deposited by evaporation, followed by annealing at 830 °C in a nitrogen atmosphere for 30 s to form the source/drain ohmic contacts. Subsequently, a 15 nm thick SiN layer is deposited by using atomic layer deposition (ALD) as the gate dielectric for the D-HEMTs. Then, the Ti/Al bilayer gate metal is evaporated for D HEMTs as well. Moreover, device isolation is accomplished through He ion implantation [25], and a 60 nm SiN passivation layer is deposited by plasma-enhanced chemical vapor deposition (PECVD). Lastly, the process concludes with two metal interconnection steps: one for connecting E/D HEMTs to form the inverter unit circuits, and another for connecting pads for testing.

**Figure 2:**
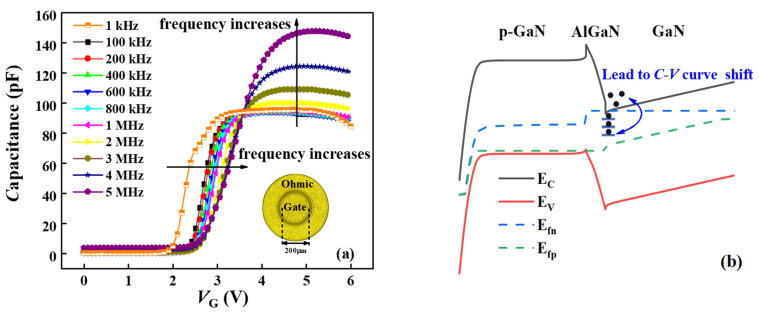
(**a**) C-V curve for 1 kHz < *f*_m_ < 5 MHz. (**b**) Schematic diagram of the mechanism of the C-V curve shift.

Capacitance–voltage (C-V) tests were conducted on the p-GaN stack capacitors across various frequencies by using an Agilent B1505A semiconductor analyzer. Systematic errors and Random errors were mitigated by calibration and by performing multiple measurements at different times and averaging the results. Figure 2a displays the C-V results across a frequency range of 1 kHz to 5 MHz. Notably, as the test frequency (*f*_m_) increases, a positive shift in the threshold voltage is observed. This phenomenon appears because increased gate voltage (*V*_G_) elevates the Fermi level above the trap level, resulting in electron capture inevitably, and consequently delays accumulation, as shown in Figure 2b. Furthermore, at higher *f*_m_, an increased number of traps at the AlGaN/GaN interface become unable to respond to rapid voltage variations, thereby further depleting channel electrons and exacerbating the C-V curve shift.

By leveraging these C-V test results, AlGaN/GaN interface trap states can be extracted through the frequency-dependent conductance method. This method measures the capacitance and conductance of the device across various frequencies to calculate the equivalent conductance (*G*_p_). As *G*_p_ reflects energy loss associated with carrier trapping and de-trapping, the characteristics of interface traps can be verified by the relationship between *G*_p_ and frequency *w* at different gate voltages, enabling further extraction of the relevant interface trap parameters [26].

**Figure 3:**
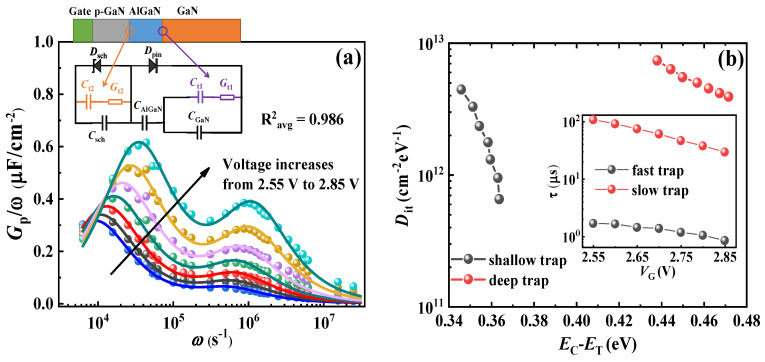
(**a**) *G*_p_/*w* is a function of *w* at selected voltages; the inset in (**a**) shows the schematic of the p-GaN gate stack and the equivalent circuit model. (**b**) *D*_it_ is a function of *E*_C_-*E*_T_ and *τ*_t_ is a function of *V*_G._

The optimized equivalent circuit of the capacitor is depicted in Figure 3a [27], which typically comprises two back-to-back diodes: a Schottky diode (*D*_Sch_) formed by metal/p-GaN contact and a PiN diode (*D*_pin_) consisting of p-GaN/AlGaN/GaN. In addition to the depletion capacitance of GaN (*C*_GaN_), interface trap states should also be considered: specifically, *C*_t1_ and *G*_t1_ (capacitance and conductance associated with AlGaN/GaN interface states), as well as *C*_t2_ and *G*_t2_ (capacitance and conductance related to p-GaN/AlGaN interface states). Meanwhile, the parallel *G*_p_/w can be derived through the following Equation (1) [27]: where *w* is the angular frequency (in s^−1^), *C*_m_ and *G*_m_ are the measured capacitance (in F) and conductance (in S), respectively. And the total capacitance (*C*_total_) should be regarded as the sum of the AlGaN barrier capacitance (*C*_AlGaN_) and the *C*_sch_, considering the contribution of *C*_t2_ and *G*_t2_ as well.

(1) $$\frac{G_{p}}{\omega }=\frac{\omega G_{m}C_{\mathrm{total}}^{2}}{C_{\mathrm{total}}^{2}+\omega^{2}{\left(C_{\mathrm{total}}-C_{m}\right)}^{2}}$$

Therefore, the *G*_p_/*w* curve at selected voltages can be calculated and plotted as dots in Figure 3a, showing a couple of peaks attributable to traps at the interface of AlGaN/GaN due to the specific bias selection. The increase and slight shift in both peaks with voltage can be attributed to the deepening of the heterojunction potential as *V*_G_ rises, making it easy for electrons to be captured by traps and implying an increase in the detected trap state density. Assuming all trap levels are single, *G*_p_/*w* can be expressed by the following, Equation (2):

(2) $$\frac{G_{p}}{\omega }=\frac{q\omega \tau_{\mathrm{ts}}D_{\mathrm{ts}}}{1+{\left(\omega \tau_{\mathrm{ts}}\right)}^{2}}+\frac{q\omega \tau_{\mathrm{tf}}D_{\mathrm{tf}}}{1+{\left(\omega \tau_{\mathrm{tf}}\right)}^{2}}$$

where *D*_ts_ and *D*_tf_ represent the slow and fast trap states for density at the AlGaN/GaN interface (in cm^−2^eV^−1^), *q* is the unit charge (in C), and *τ*_ts_ and *τ*_tf_ are the time constant of the slow and fast trap states (in μs) [26,27].

Based on Equation (2), the *G*_p_/*w* can be fitted to align with calculated data. The fitting results show great agreement, with an average R-square (R^2^_avg_) of 0.986, which is helpful to obtain relatively accurate trap state parameters: *D*_ts_ = 3.93 × 10^12^~7.4 × 10^12^ cm^−2^eV^−1^, *τ*_ts_ = 29.6~108 μs; *D*_tf_ = 6.55 × 10^11^~4.45 × 10^12^ cm^−2^eV^−1^, *τ*_tf_ = 0.85~1.69 μs. To extract the trap activation energy *E*_C_-*E*_T_, *τ*_t_ is determined by using the following, Equation (3) [26,27]:

(3) $$\tau_{t}=\frac{1}{v_{\mathrm{th}}\sigma_{n}N_{c}}\exp\left(\frac{E_{C}-E_{T\;}}{kT}\right)$$

where *v*_th_ is the average thermal velocity (in cm/s), *σ*_n_ is the electron capture cross-section (in cm^−2^), *T* is the temperature (in K), *E*_C_ is the conduction band energy, *E*_T_ is the trap level energy, *E*_C_-*E*_T_ is the trap activation energy (in eV), and *N*_c_ is the effective state density of the GaN conduction band (in cm^−3^). In this study, *T* = 300 K, *v*_th_ = 2.6 × 10^7^ cm/s, *σ*_n_ = 1 × 10^−14^ cm^−2^, and *N*_c_ = 2.7 × 10^18^ cm^−3^ [28].

The *D*_it_ as a function of *E*_C_*-E*_T_ and τ_t_ as a function of gate voltage (*V*_G_) are shown in Figure 3b. The activation energy *E*_C_*-E*_T_ of the shallow level at the AlGaN/GaN interface ranges from 0.345 eV to 0.363 eV, while the deep level ranges from 0.438 to 0.471 eV. The shallower trap is generally consistent with 0.33 to 0.40 eV, detected from the traditional HEMTs that are normally on. The status of these original defects is probably oxygen-related [27,29]. The deeper trap at this energy level in the AlGaN/GaN interface can be found in the previous studies as well [27,30,31]. Obviously, of the two trap states, the shallower trap exhibits a larger state density, with the time constant decreasing as *V*_G_ increases.

**Figure 4:**
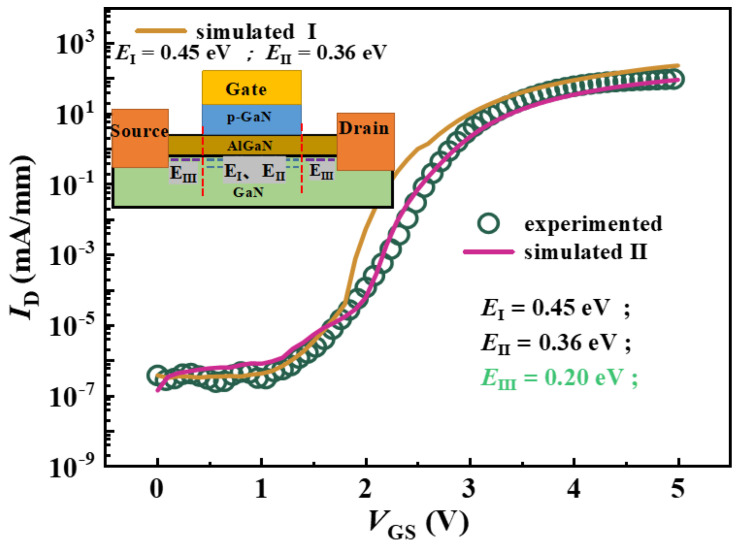
Transfer characteristics of p-GaN HEMT.

Using the extraction results, the transfer characteristic of p-GaN HEMT is fitted to validate the TCAD simulation models. The simulation incorporates physical models, including the trap model, field-dependent mobility model, SRH model, and impact ionization model [32]. The fitting results are shown in Figure 4.

When only two acceptor traps are introduced at the AlGaN/GaN interface (trap activation energy *E*_I_ = 0.45 eV and *E*_II_ = 0.36 eV), the simulated transfer characteristic curve is basically consistent with the experimental data in the sub-threshold region. This agreement suggests that the variation in the premature-on stage primarily results from the deep-level traps at the AlGaN/GaN interface [33]. However, when *V*_GS_ > 1.5 V, the simulated current exceeds the experimental current due to the neglect of the shallow acceptor trap. Thus, by incorporating the shallower acceptor trap (*E*_III_ = 0.20 eV) in the access region, which has been reported in other works and been suggested to be related to, for example, N vacancies or interface states [34,35,36], the simulation result is in accordance with the experimental data.

## 3. Results and Discussion

**Figure 5:**
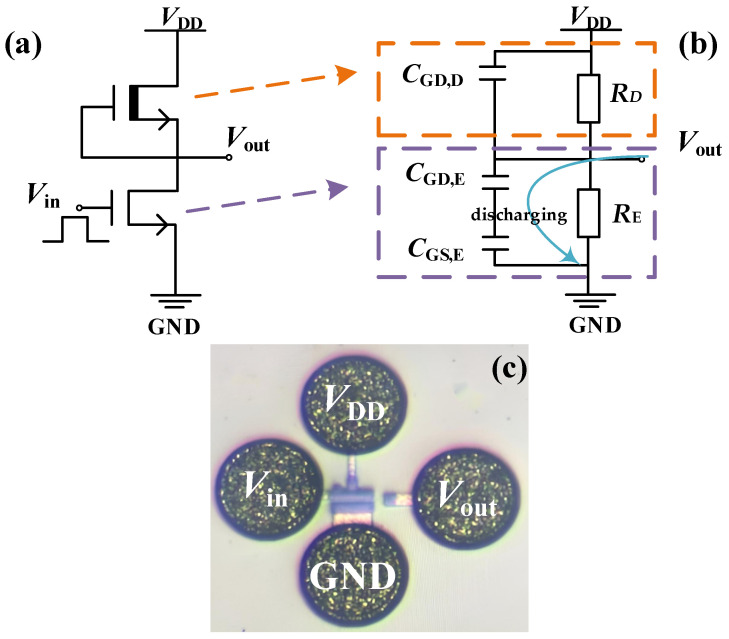
(**a**) The circuit of the DCFL inverter. (**b**) The equivalent discharge circuit of the DCFL inverter, (**c**) The microscope image of the DCFL inverter.

The DCFL inverter circuit and its microscope image are depicted in Figure 5a,c, with the p-GaN HEMT’s drain connecting to the MIS-HEMT’s source and gate to form the output. The DCFL inverters the transmit and switch logic signals through the p-GaN HEMTs, regarded as switching transistors. In this case, the switching transistors undergo obvious voltage and current changes during the logic switching, so the interface traps notably affect their threshold voltage and on-resistance, which directly determines the low-level voltage (*V*_OL_) and switching speed. Conversely, the load transistors function as stable current sources with relatively fixed operating conditions, where trap effects minimally impact inverters’ performances. Therefore, the switching transistor’s trap effects are mainly analyzed to assess their leading role in DCFL inverters’ turn-on characteristics.

### 3.1. Influence of V_DD_ on Inverter’s Performances

**Figure 6:**
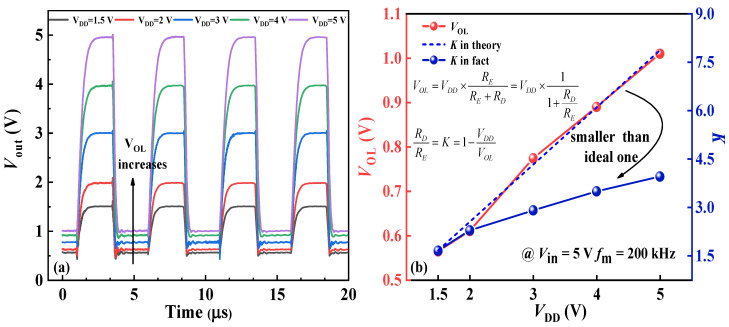
(**a**) Output voltage *V*_out_ waveforms diagram under different *V*_DD_. (**b**) Low-level voltage (*V*_OL_) and *K* value under different *V*_DD._

In the experiment, the Tektronix AFG31000 arbitrary function generator (Shanghai, China) was used to offer the input voltage *V*_in_ with the supply voltage offered by the DC power source, and observed the output voltage *V*_out_ waveform through the Tektronix MSO54B oscilloscope (Shanghai, China). Figure 6a displays the output voltage (*V*_out_) waveforms of the inverter across various *V*_DD_ values at an operating frequency of 200 kHz, with *V*_in_ set at 5 V to fully activate the switching transistor and stabilize *V*_OL_. As *V*_DD_ rises from 1.5 V to 5 V, the inverter consistently achieves a high-level voltage *V*_OH_ = *V*_DD_. However, *V*_OL_ varies across *V*_DD_ values, contrary to typical reports of *V*_OL_ stability [11,12]. Instead, as *V*_DD_ increases, *V*_OL_ rises from 0.56 V to 1.01 V, growing by 80%.

When the switching transistor is activated, the original high-level output voltage (*V*_OH_) decreases to the low-level output voltage *V*_OL_ via the discharging circuit, as illustrated in Figure 5b [37]. Here, *C*_GD,D_, *C*_GD,E_, *C*_GS,E_, *R*_E_, and *R*_D_ represent the gate-to-drain capacitance of the load transistor, the gate-to-drain capacitance of the switching transistor, the gate-to-source capacitance of the switching transistor, and the resistance of switching or load transistor, respectively. Thus, when the inverter reaches stability, *V*_OL_ can be interpreted as *V*_DD_ shared by the switching and load transistors, expressing their resistance ratio *R*_D_/*R*_E_ in terms of *V*_DD_ and *V*_OL_, defined as parameter *K*. As shown in Figure 6b, the measured K value is lower than the theoretical value, suggesting that a higher *V*_DD_ enlarges the on-resistance of the switching transistor.

**Figure 7:**
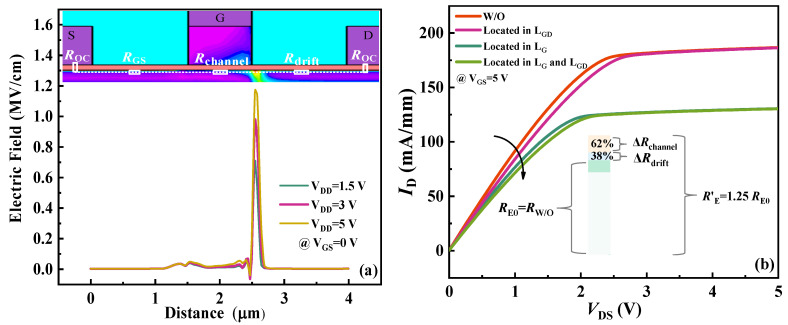
(**a**) Simulated channel electric field density under different *V*_DD;_ (**b**) impact of trapping location on resistance degradation.

In Figure 7a, simulations illustrate that the electric field at the switching transistor’s gate/drain edge intensifies with increasing *V*_DD_, promoting electron trapping. This phenomenon results in a positive shift in threshold voltage, elevated on-resistance, and an increase in *V*_OL_. Figure 7b further simulates the impact of trapping on the switching transistor’s on-resistance at different interface regions, specifically the drain access region *L*_GD_ and the gate region *L*_G_. In the absence of traps, the switching transistor’s on-resistance can be represented as a series of contact resistance *R*_OC_, source-channel resistance *R*_GS_, drift-region resistance *R*_drift_, and channel resistance *R*_channel_, collectively denoted as *R*_E0_ or *R*_W/O_. When traps are present at *L*_GD_ or *L*_G_, they incrementally add Δ*R*_drift_ or Δ*R*_channel_ to the total on-resistance. The results demonstrate that trapping in the gate region is a primary contributor, accounting for 62% of the total on-resistance degradation, whereas trapping in the drain access region contributes 38% and has minimal effect on the saturated drain current.

### 3.2. Influence of f_m_ on Inverter’s Performances

**Figure 8:**
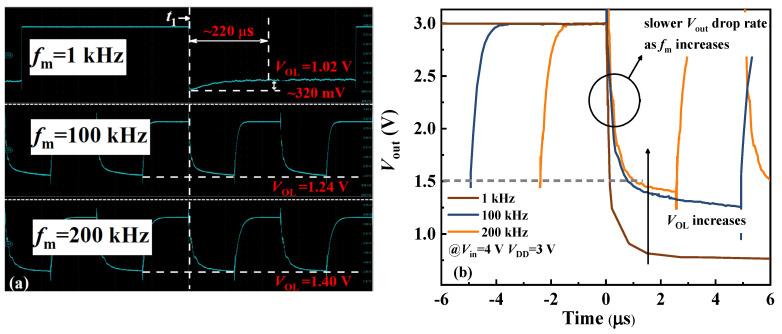
(**a**) Oscilloscope screenshot of *V*_out_ waveforms at different frequencies. (**b**) Comparison diagram of falling edge at time t_1._

Figure 8a presents the *V*_out_ waveforms of the GaN DCFL inverter at different operating frequencies (*V*_in_ = 4 V, *V*_DD_ = 3 V). The inverter can achieve *V*_OH_ = *V*_DD_ across all frequencies. However, a comparison of the falling edge at time t_1_ reveals that as the operating frequency increases from 1 kHz to 200 kHz, *V*_OL_ rises from 1.02 V to 1.40 V. The time required for *V*_out_ to decrease from *V*_DD_ to 0.5 *V*_DD,_ defined as *t*_fall_, increases from 153 ns to 1 μs as the frequency rises from 1 kHz to 200 kHz, which is shown in Figure 8b. The falling time *t*_fall_, based on the generic textbook and related articles, can be expressed using Equation (4) [13], where *C*_GD,E_, *μ*_n,E_, *C*_G,E_,${\;(\frac{L}{W})}_{E}$, *V*_GS,_ and *V*_TH, E_ represent the gate-drain Miller capacitance (in F), electron mobility (in cm^2^/V‧s), gate capacitance (in F), width–length ratio, gate-source voltage (in V), and threshold voltage of the switching transistor (in V), respectively.

(4) $$t_{\mathrm{fall}}=\frac{C_{\mathrm{GD},\;E}}{\mu_{n,E}C_{G,E}}{\left(\frac{L}{W}\right)}_{E}\frac{V_{\mathrm{DD}}}{{\left(V_{\mathrm{GS}}-V_{TH,E}\right)}^{2}}$$

It can be observed from the formula that at a given temperature, the falling time mainly depends on *V*_TH,E_. In the previously mentioned *C*-*V* test, it was found that as *f*_m_ increases, the extent of interface trapping intensifies, leading to a gradual increase in *V*_TH, E_ and rising on-resistance *R*_E_. This degradation in conduction raises *V*_OL_, with increasing discharging RC delay and reduced transmission speed. Consequently, within the same period, *V*_out_ cannot reach its previous value, causing a rise in *t*_fall_.

**Figure 9:**
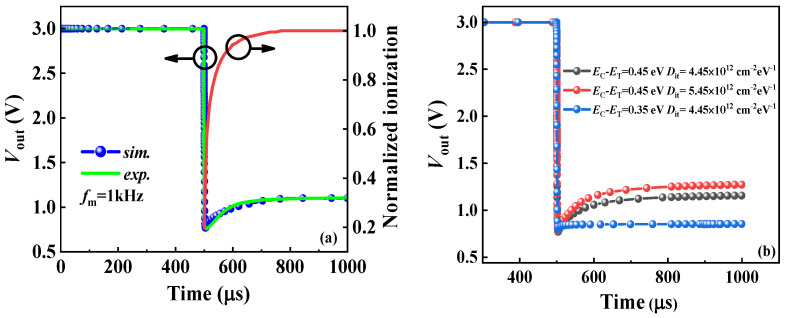
(**a**) Numerical fitting of the output waveform of the inverter at fm = 1 kHz and the interface state ionization rate after turn-on. (**b**) The impact of trap parameters on the inverter’s performance.

Additionally, at the low frequency of *f*_m_ = 1 kHz, *V*_out_ does not immediately reach a stable *V*_OL_ upon turn-on, but instead shows a voltage swing of approximately 220 μs and 320 mV. Based on the modified TCAD models, *V*_out_ at an operating frequency of 1 kHz is numerically analyzed. As depicted in Figure 9a, the simulation results show good agreement with experimental data when combined with interface state information extracted via the frequency-conductance method before. By comparing the ionization rate of the interface state (normalized ionization) at different moments, it was found that the rise in *V*_OL_ after turn-on is consistent with the increase in normalized ionization. This suggests that at low frequencies, captured electrons are more readily released from traps, contributing to conduction with a faster response speed than at 100 kHz and 200 kHz, thereby achieving a lower *V*_OL_ and showing the delay phenomenon. Meanwhile, the impact of trap parameters on the inverter’s performances is evaluated through simulation. As shown in Figure 9b, higher interface trap density further depletes channel electrons [33], raising *R*_channel_ and *V*_OL_ as a result. Additionally, deeper trap energy levels cause a more severe shift and longer switching delay [38].

According to the above results, the observed performance degradation of DCFL inverters, caused by interface trapping at the AlGaN/GaN interface, has significant implications for GaN-based power device design in industrial applications. As supply voltage and operating frequency rise, threshold voltage shifts and increased fall times can severely affect the switching speed and efficiency of GaN power circuits. In high-frequency applications like power converters or digital logic circuits, this degradation can result in higher power consumption, slower response times, and potential instability in logic operations. These findings highlight the need to address interface trap issues in GaN devices to optimize their performances in real-world power-switching circuits. Therefore, better interface engineering is required to foster device dynamic performances [29]. Device engineers must consider the dynamic behaviors of interface traps and safety margins when designing GaN devices and circuits to ensure reliable operation under varying voltage and frequency conditions.

## 4. Conclusions

In summary, the impact of interface traps at the AlGaN/GaN interface on the p-GaN HEMT DCFL inverter’s performance under varying *V*_DD_ and *f*_m_ is investigated. Unlike conventional studies primarily focused on temperature-related effects, the significance of voltage and frequency as additional factors influencing the GaN inverter’s performance is emphasized. Two trap states located at the AlGaN/GaN interface with *E*_C_-*E*_T_ range from 0.345 eV to 0.363 eV and 0.438 to 0.47 eV. The experiment results indicate that an increase in *V*_DD_ elevates the electric field at the switching transistor’s gate/drain edge, which enhances electron trapping and subsequently raises the threshold voltage, on-resistance, and low-level output voltage. As *V*_DD_ increases from 1.5 V to 5 V, *V*_OL_ rises from 0.56 V to 1.01 V. Additionally, at higher *f*_m_, interface traps fail to respond effectively to rapid voltage changes and further deplete channel electrons, resulting in threshold voltage shift and increased fall time, which consequently reduces the switching speed. As *f*_m_ increases from 1 kHz to 200 kHz, *V*_OL_ rises from 1.02 V to 1.40 V, with the fall time increasing from 153 ns to 1 µs. Overall, these findings advance our understanding of limitations imposed by interface traps on GaN DCFL inverter efficiency. Compared to conventional GaN inverters reported in the literature, the proposed analysis establishes the relationship between the trapping mechanism and GaN unit circuit degradation, offering a pathway to optimizing future GaN-based power circuits. Future research should explore the combined impact of temperature, *V*_DD_, and *f*_m_ on trap dynamics to enhance understanding and better optimize GaN device designs for high-power and high-frequency applications.

## Data Availability

Data are contained within the article.
